# Effects of Universal School-Based Parental Support for Children’s Healthy Diet and Physical Activity—the Healthy School Start Plus Cluster–Randomised Controlled Trial

**DOI:** 10.1007/s11121-024-01697-4

**Published:** 2024-07-11

**Authors:** Åsa Norman, Mahnoush Etminan Malek, Gisela Nyberg, Emma Patterson, Liselotte Schäfer Elinder

**Affiliations:** 1 Department of Global Public Health, Karolinska Institutet, 171 77 Stockholm, Sweden; 2 Department of Clinical Neurosciences, Karolinska Institutet, Nobels Väg 9, 171 65 Solna, Sweden; 3 The Swedish School of Sport and Health Sciences, Lidingövägen 1, 114 33 Stockholm, Sweden; 4 Section for Risk and Benefit Assessment, Swedish Food Agency, Box 622, 751 26 Uppsala, Sweden; 5 Centre for Epidemiology and Community Medicine, Region Stockholm, 104 31 Stockholm, Sweden

**Keywords:** BMI, Intervention, Motivational interviewing, Obesity, Parenting, Prevention, School health care, Sweden, Type 2 diabetes

## Abstract

Health promotion from an early age is key to preventing unhealthy weight development in childhood, and parental involvement is essential. The school-based Healthy School Start intervention aims to promote healthy dietary and activity habits in the home environment and prevent child obesity through parental support. This study evaluated the effectiveness of the third iteration of the programme on children’s dietary and activity behaviours, and body composition through a cluster-randomised controlled trial. The trial included 17 schools (8 intervention) in disadvantaged areas in mid-Sweden with 353 families with 5- to 7-year-old children. The primary outcomes were intake of selected healthy and unhealthy foods and beverages measured using photography. Secondary outcomes were physical activity and sedentary time measured by accelerometry, and measured weight and height. All outcomes were assessed at baseline and post-intervention (8 months). Linear multi-level regression showed significant favourable effects of the intervention for intake of sweet beverages (*b* =  − 0.17, *p* = 0.04), intake of healthy foods (*b* = 0.11, *p* = 0.04), and more time in moderate to vigorous physical activity during weekdays (*b* = 5.68, *p* = 0.02). An unfavourable sub-group effect of the intervention was found for children from families with low education regarding sedentary time on weekends (*b* = 23.04, *p* = 0.05). The results align with the previous two trials of the programme, indicating that school-based parental support is a useful approach for health promotion in young children in disadvantaged areas. Trial registration: ClinicalTrials.gov: No. NCT03390725, retrospectively registered on January 4, 2018, https://clinicaltrials.gov/ct2/show/NCT03390725.

**Supplementary Information:**

The online version contains supplementary material available at 10.1007/s11121-024-01697-4.

Social inequalities in health resulting from unhealthy diets and low physical activity is a key challenge to public health not only in the Nordic countries (Eikemo & Øversveen, 2019) but throughout all countries in The Organization for Economic Cooperation and Development (OECD) (Bleich et al., 2012). An unhealthy diet and lack of physical activity are among the most important risk factors for the disease burden in Sweden and globally (Gakidou et al., 2017), including obesity and other chronic diseases. A rapid increase in childhood obesity occurred in the Nordic countries and worldwide in less than one generation (Lobstein et al., 2015). Recent data from the Swedish Public Health Agency show that the rise continues in all age groups and fastest in the younger age groups (Public Health Agency of Sweden, 2022). Today, the prevalence of overweight and obesity is around 23% in children aged 6–9 years in Sweden and large social inequalities persist (Public Health Agency of Sweden, 2023). Social inequalities in health can be defined as the systematic, avoidable, and unfair differences in health outcomes that can be observed between populations, between social groups within the same population, or as a gradient across a population ranked by social position (McCartney et al., 2019). In younger children in Sweden, this inequality can be seen in dietary habits, which are shown to be less healthy in families with low education (Elinder et al., 2014) and where parents are not originally from the Nordic region (Safsten et al., 2016). Schools may have an important compensatory effect where school meals are on average more nutritious than non-school meals (Eustachio Colombo et al., 2020). There is also evidence to suggest that children are less physically active at home/during weekends (Comte et al., 2013; Nyberg et al., 2009, 2015, 2016).

Schools serve as an important and effective setting for health promotion and obesity prevention programmes when both diet and physical activity components are included (Bleich et al., 2018; Gori et al., 2017). However, the home environment is also critical for children’s development of healthy habits (Gori et al., 2017; Sisson et al., 2016; Yee et al., 2017). Several systematic reviews have concluded that childhood obesity prevention interventions are more effective when parents are actively involved (Brown et al., 2019; Gori et al., 2017; Sisson et al., 2016; Skouteris et al., 2011; Ward et al., 2017). Many parenting practices and parent–child interactions shape children’s health-related behaviours (Yee et al., 2017). Making healthy food available in the home, serving as role models for healthy eating, and active and restrictive guidance show strong associations with healthy food consumption (Yee et al., 2017). Logistic support and role modelling have been associated with higher physical activity in children (Xu et al., 2015). In addition, lack of parental cooperation regarding the promotion of healthy habits at home and negative parent–child interactions may act as barriers to healthy behaviours (Norman et al., 2014).

Starting in 2010, the Healthy School Start (HSS) programme was developed to be carried out in the school context in Sweden, universally targeting children attending the first, preparatory year in school (at 5–7 years of age), and their parents. The HSS has previously been tested in two cluster-randomised controlled trials in areas with mixed socioeconomic position (SEP) (Nyberg et al., 2015) and low SEP (Nyberg et al., 2016). These studies found favourable effects, primarily on dietary intake (Nyberg et al., 2015, 2016), and significantly reduced BMI among children with obesity at baseline (Nyberg et al., 2016). Interviews with parents and teachers as part of the process evaluations showed that parents found the HSS compatible with their day-to-day life, and the intervention materials were perceived by teachers as structured and easy to use. Elements of the HSS that could be improved were identified, such as additional tailoring of intervention activities to parental abilities, activities to increase parental engagement in the programme, an increased focus on parenting behaviour, and cooperation within the family and between the home and the school (Bergstrom et al., 2015; Norman et al., 2014, 2016, 2019).

Based on the findings from the first two trials, the programme was revised with an increased focus on parenting practices combined with information on healthy habits included in the first two trials. Also, an additional component, the FINDRISC test of type 2 diabetes risk (Saaristo et al., 2005) was included to increase health awareness in families at higher risk and increase parental engagement. In contrast to the previous trials, where the motivational interviewing (MI) component was performed by external personnel with high MI competence, in this trial the school nurse carried out the MI to increase the likelihood of the programme becoming sustainable. The objective of this study was to compare the effectiveness of The Healthy School Start Plus (HSSP) intervention to standard care and additional health information, on outcomes related to diet, physical activity, and weight development of children starting school in disadvantaged areas with higher health needs.

## Methods

### Study Design, Randomisation, Setting, and Participants

**Fig. 1:**
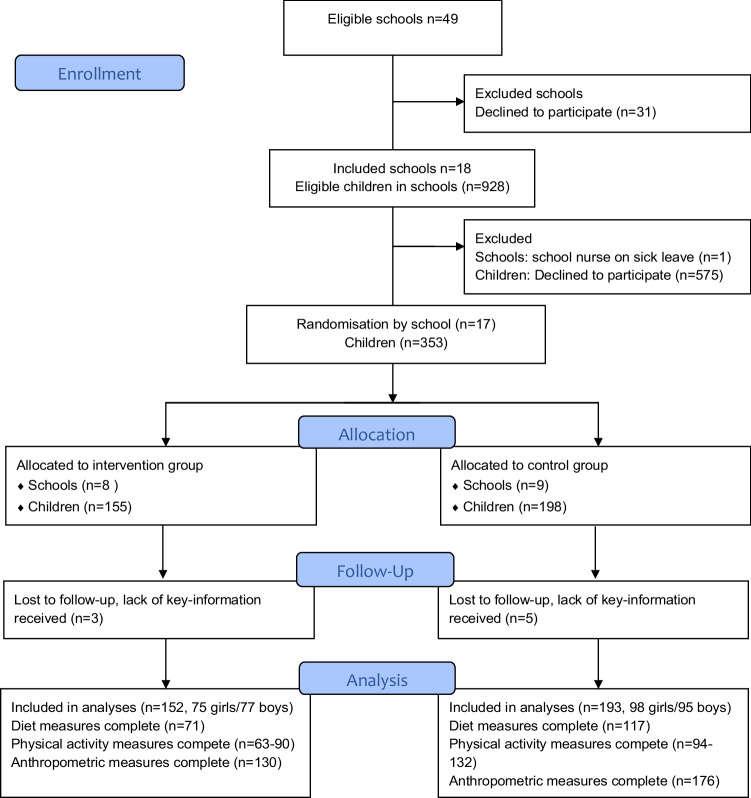
CONSORT 2010 flow diagram

The HSSP was carried out as a cluster-randomised, two-arm parallel trial with a wait-list control group and is reported according to the CONSORT checklist (Supporting Information 1), and the TIDieR Checklist (Supporting Information 2). Randomisation was conducted by an external statistician who used a computer-generated randomisation procedure to randomise schools with a 1:1 allocation ratio. Blinding was applied to all parents, children, teachers, and research staff until baseline measurements were completed, but was revealed to school nurses, principals, and the project leader at the time of randomisation to allow school nurses in the intervention group to undergo training in MI prior to the start of the intervention. Parents in control schools received the health information brochure (component 2 below) and were offered the remaining intervention components after completion of the last outcome measurement in June 2019. Recruitment for the study was carried out during January–May 2017 and has been described in detail in the study protocol (Elinder et al., 2018). Recruitment was undertaken in two steps: first, schools were invited, and second, parents were invited. Inclusion criteria on school level were that the school was a primary school, that the proportion of parents with university education was lower than 50 percent, and that the school was situated in mid-Sweden. In total, 49 schools were invited. The inclusion criterion for parents was that they had a child in pre-school class in one of the participating schools. Initially, 18 schools agreed to participate in the study, but one dropped out before recruitment of parents had started as the school nurse went on sick leave (see Fig. 1). In the end, 17 schools and 353 families with a child in pre-school class consented to participate in the study. Among the schools that agreed to participate, the average proportion of parents with university education was 37% compared to the Swedish average of 57%. Schools were situated in seven different counties in mid-Sweden in areas with relative disadvantage. The average proportion of parents born outside of Sweden was 47% among the participating schools compared to the average of 20% in Sweden.

### The Healthy School Start Plus Intervention

The HSSP aims to promote healthy dietary and physical activity behaviours in the home environment and prevent unhealthy weight development among children starting school through structured school-based parental support. The HSSP is based on social cognitive theory (Bandura, 1986), with four components that target mediators on parental level (self-efficacy, behavioural capability, outcome expectations and expectancies, observational learning, motivation), and child level (motivation) to subsequently affect outcomes on child level. The HSSP is described in detail in a study protocol (Elinder et al., 2018). The intervention components comprise:

- Motivational interviewing (Miller & Rollnick, 2013) with parents targeting parental self-efficacy, behavioural capability, outcome expectations and expectancies, role modelling, and motivation: Parents (one or both) are invited by the school nurse to a face-to-face MI session without the presence of the child. This is an extension of the routine first-school-year health visit and takes about 15–30 min. The aim is to explore potential behaviour changes, or sustain pre-existing healthy behaviours, in the home environment that are related to the child’s dietary, and/or activity behaviours. The parents themselves choose the focus of the conversation. To assist the nurses in conducting the MI sessions, nurses follow a flexible template with, e.g., suggested introductions of topics and open-ended questions, based on the four processes of MI. This template was developed by research staff with clinical experience of conducting MI and members of the Motivational Interviewing Network of Trainers (MINT). Some schools offer parents a second school visit as part of their usual routine if necessary. Before the start of the intervention, school nurses in the intervention group attended a 2-day training session performed by MINT trainers. Training included supervised audio-recorded sessions using the Motivational Interviewing Treatment Integrity Code protocol 4.2 (MITI) (Moyers et al., 2016) and corrective feedback. During the intervention, nurses received corrective feedback on audio-recorded MI sessions with parents on one to four occasions (median = two occasions). Control group nurses were offered the MI training after the intervention.
- Health information to parents targeting parental behavioural capability: Parents receive an intervention-specific brochure containing evidence-based information on parenting in relation to children’s healthy dietary and physical activity behaviours, and age-appropriate recommendations for diet, physical activity, screen time, and sleep.
- Classroom lessons for children and home assignments for children and parents jointly: The lessons target child motivation. Teachers use an intervention-specific teacher’s manual to conduct nine activity-based lessons on diet, physical activity, screen time, and sleep. Each lesson is accompanied by a home assignment to be completed in a workbook with the support of a parent. The home assignments targeting parental self-efficacy and behavioural capability include instructions for parents on how to carry out the assignment and provide an opportunity for parents to practice the positive parenting practices as described in the brochure.
- Type 2 diabetes (T2D) risk test for parents to theoretically target parents’ motivation and awareness of health for the whole family including their own health behaviours. Parents complete the web-based FINDRISC test (Saaristo et al., 2005) and receive automated feedback on their level of risk for developing T2D. In case of medium or high risk, the parent is instructed to consult a primary health care centre for health counselling according to standard routines.

Children in the control schools received treatment as usual according to prevailing guidelines for school health services and in addition all parents received the same health information brochure as the intervention group.

### Implementation Strategies

To facilitate implementation in schools, and to encourage active parental participation, four specific strategies were applied (Elinder et al., 2018) and named according to the SISTER taxonomy (Cook et al., 2019): (1) Obtain formal commitments (a written agreement specifying school and researcher obligations was drawn up); (2) distribution of educational material (a website with information and video instructions for teachers was created); (3) ongoing MI training for nurses; and (4) prepare families and facilitate school-parent collaboration through a teacher-led kick-off meeting.

### Data Collection

#### Outcome Data

Outcome data at individual level were acquired at baseline (T0) in September–October 2017, and after intervention (T1) in April–May 2018.

##### Children’s Dietary Intake – Primary Outcome

The primary outcome was the average intake per week of selected foods and drinks; the intake of which is generally to be either encouraged or limited. The individual foods and beverages were summed to create three outcomes, termed “healthy foods” (fruits, vegetables, and legumes), “unhealthy foods” (crisps, salty snacks, sweets, chocolate, cakes, biscuits, desserts, and other sweet foods), and “sweet beverages” (soft drinks, flavoured milk, and all kinds of fruit juice). To measure intake, we developed a mobile phone photography–based method specifically for this study, with the aim of minimising both participant recall error and reporting burden (Norman et al., 2020). This method was chosen over parent-reported questionnaires as the photography-based method had a higher response rate than the questionnaires and was found acceptable in a validation study (Norman et al., 2020). The validation study showed that while under-reporting was common, it was relatively constant at all levels of intake, and the method was adequate for group comparisons. Briefly, parents were instructed to photograph all food and drink consumed outside of school hours on two weekdays and one weekend day, before and after each eating occasion. They used their mobile phone camera and sent pictures by MMS (text was optional). Photos were briefly reviewed in near real time and in case of poor quality, a clarifying question was sent. A question at the end of each day was sent to check if the day’s reporting was complete, but contact was otherwise kept to a minimum. Parents were assigned to take photos either Monday, Wednesday, and Saturday or Tuesday, Thursday, and Sunday. If the child was ill or on holidays, the dates were rescheduled. From the data sent in, trained coders estimated the volume (in deciliters) of the selected foods and drinks provided, and the percentage consumed. The coders followed a standardised protocol and consulted a specially developed library of reference photos of common foods in various states and portions. Coders worked independently and inter-rater reliability was monitored using a subsample of 17 participants coded (independently) in triplicate. Using an absolute agreement, 2-way mixed-effects model, ICC for all three outcomes was “excellent” (lower bound 95% CI > 0.9) at T0, and “very good” to “excellent” (lower bound 95% CI > 0.83) at T1. To be included in the analysis, data from at least one weekday (96% had two) and one weekend day was required at both T0 and T1. No minimum intake was required for inclusion.

##### Children’s Physical Activity and Sedentary Time – Secondary Outcome

Physical activity and time spent sedentary were measured with accelerometers (GT3X + , Actigraph, LCC, Pensacola, USA) which is considered valid and reliable (de Vries et al., 2006). The children wore the accelerometers on the right hip for seven consecutive days during all hours awake except for activities involving water. ActiLife Data Analysis, version 6.13.3 was used to process the accelerometer data saved in 5-s epochs at a sample rate of 30 Hertz. Non-wear time was defined as 60 min of consecutive zeros allowing for 2 min of non-zero interruptions. A day was considered valid when the accelerometer was worn for at least 500 min. For the week to be valid and included in the analysis, at least 500 min of activity registration per day for a minimum of two weekdays days were required for weekdays, and at least one weekend day in the analysis regarding weekends. A time filter was set between 07.00 and 22.59. A separate time filter was used for non-school time between 07.00 and 07.59 and 16.00 and 22.59. Cut-offs were used to estimate sedentary activity (0–100 counts/min) and moderate to vigorous physical activity (MVPA) (≥ 2296 counts per minute) (Evenson et al., 2008; Trost et al., 2011). A weekly average of ≥ 60 min of MVPA per day was used to categorise the participants into reaching or not reaching the physical activity recommendation.

##### Children’s Anthropometry – Secondary Outcome

Children’s height, weight, and waist circumference were measured by trained assistants, using SECA instruments, according to standardised procedures (Elinder et al., 2018) to the nearest 1 mm for height and waist circumference, and 100 g for weight. BMI was calculated (kg/m^2^) and a standard deviation score (BMI-sds) was calculated according to a Swedish reference standard (Karlberg et al., 2001). Weight status (underweight, normal weight, overweight, and obesity) was classified according to International Obesity Task Force (Cole & Lobstein, 2012).

##### Level of Education and Region of Birth

The initial intention, described in the study protocol, was to develop an index for socioeconomic position based on parental level of education and occupation. However, data on occupation turned out to be difficult to interpret (missing or unclear text data) and we therefore defaulted to using only parental education, in line with the previous trials. If at least one parent-reported education > 12 years, the family was classified as having a “high level of education” (otherwise “low”). Where both parents reported their country of birth as Sweden, Norway, Denmark, Finland, or Iceland, the family was considered to be “Nordic” (otherwise “non-Nordic”).

### Process Data

Process measurements collected in the trial were overall fidelity to the intervention components (if the components were delivered as intended) and included the following measures (Moore et al., 2015): adherence (delivery of the health brochure, MI, and T2D risk test), dose (the quantity of classroom lectures delivered), and quality of the MI component. Regarding adherence to the health brochure and MI sessions, all parents received the brochure, but adherence is reported as the proportion of parents who participated in the MI sessions. The nurse noted whether the parents had received and read the brochure and attended the MI session. Adherence to the T2D risk test was objectively monitored as parents completed the test electronically. Dose of the classroom lessons was assessed using a logbook where teachers indicated the time spent on lessons, the number of completed core elements, and home assignments. The quality of the MI delivery was assessed by audio recording of all intervention MI sessions. Recordings were coded by reliable coders from the MIQA group coding lab at Karolinska Institutet using the MITI 4.2 instrument (Moyers et al., 2016). In addition, differences in MI competence between nurses in the intervention and control group were assessed before and after the intervention group nurses had received the MI training. This test was carried out as some school nurses had attended MI training prior to the HSSP, and as MI counselling behaviour can be a more or less natural way of conducting conversations. The test was done by having all nurses conduct an audio-recorded MI session with an actor posing as a standardised parent character.

### Statistical Analysis

Descriptive data are presented as means and standard deviations. Group differences at baseline were examined using chi-square test for categorical data, and independent samples *t*-test and the Mann–Whitney *U* test for continuous data. Linear mixed-effect regression analysis with two levels (individual and school) was used to compare differences in outcome at T1 between the intervention and control group. Effects were first tested using fixed effects only, after which a random intercept on level 2 (school) was added to adjust for clustering on school level. Model fit was assessed between models with fixed effects and with an added random intercept by comparing to − 2 Log Likelihood values. The following three models were used: model 1, a crude model which was first tested on all outcomes at T1 with group (intervention/control) as the predictor with adjustment for baseline values of the respective outcome. In model 2, child sex and parental education were added to model 1. Here, effects were tested for moderation by child sex and parental education by including interaction terms for exposure*child sex, and exposure*parental education. If significant interaction terms were found, the analysis was stratified. In the previous two HSS intervention trials, parental region of birth was explored as a moderator of intervention effects. In order to enable comparison between all three HSS trials, region of birth was tested in a third model in the present study, despite not being described in the study protocol. Thus, in model 3, child sex and parental region of birth were added to model 1 and effects were tested for moderation by child sex and parental region of birth by including interaction terms for exposure*child sex, and exposure*parental region of birth. Again, if significant interaction terms were detected, the analysis was stratified. Physical activity outcomes (time in MVPA and spent sedentary) were adjusted for accelerometer wear time. Sedentary time (weekdays, weekend, and non-school time) was also adjusted for MVPA during the same period. Regarding dietary variables, logarithmic transformation (LOG10 (X + 1)) was performed to improve normality, linearity, and homoscedasticity. Regarding BMI-sds, stratified analyses on weight status categories were performed to explore effects on children with overweight and obesity specifically. Analyses were conducted in the following steps:

1. Complete cases intention to treat (ITT) analysis, where all children who had agreed to participate and had valid data at T0 and T1 were included in the analysis. This is the main analysis and is presented in text and tables.
2. Analyses per protocol included children in the intervention group whose parents participated in the MI session of the intervention (hypothesised to be the most influential component for which participation was possible to fully monitor) compared to children in the control group for each outcome (*n* = 27 to 184 depending on the outcome).
3. A sensitivity analysis using last value carried forward (LVCF) was performed for outcomes and models showing a significant intervention effect. We chose to use LVCF instead of multiple imputation as the LVCF approach can be regarded as more conservative and reduces the risk of type 1 error. The sensitivity analyses included all children who had valid data at baseline for each outcome (*n* = 218 to 342 depending on the outcome).

The a priori power calculation was based on detecting a difference in intake of unhealthy foods of 0.8 portions/day between the groups. With a power of 80%, a significance level of 5%, and an intra-class correlation coefficient of 2% (allowing for clustering at school level), the sample target was 17 schools with 15 children participating in each school, 255 children in total (Elinder et al., 2018).

Process data were analysed using descriptive statistics and reported as means (SD), and proportions. In order to assess if nurses in the intervention group had a higher MI competence than nurses in the control group, differences in MI competence between the groups were tested using independent sample *t*-tests. MI quality is presented as group means for important categories of MI counsellor behaviour as coded by the MITI 4.2 (Moyers et al., 2016). These categories are the technical (counsellor cultivating client change talk and softening client sustain talk) and the relational (counsellor seeking partnership and expressing empathy) components, ratio of counsellor-expressed reflections and questions, and overall MI-adherent and non-adherent counsellor behaviour (Moyers et al., 2016). All statistical analyses were performed using the software IBM SPSS, version 26.0 (Chicago, Illinois, USA), and the level of significance was set to 0.05.

## Results

### Outcome Evaluation

Nine children dropped out of the study (Fig. 1). These children came from eight different schools and did not differ from the children included in the analyses regarding sex. Descriptive data and group differences at baseline for the whole sample and complete cases included in the analyses are shown in Table 1. Although arising from chance due to the randomisation procedure, we noted that the proportion of non-Nordic families and time spent sedentary during weekends was significantly higher in the intervention group, and the proportion of families with low education was higher in the control group at baseline for the whole sample. A similar patten was seen in the complete cases. Of the total sample, 36.8% of the families had a low level of education and 51.3% were non-Nordic, of whom most were born in Iraq, Syria, Turkey, Eritrea, Iran, or Afghanistan. The results of the primary analysis of intervention effectiveness are described in the text and in Tables 2 and 3. An exploration of families who dropped out of the intervention showed that families with parents of a lower education level or who originated from outside of the Nordic region dropped out to a significantly higher extent than parents with higher education or from the Nordic region.

**Table 1:** Descriptive characteristics of children at baseline in intervention and control group

			*Total sample*					*Sample in complete case analysis*			
			Total	Intervention	Control	*p*	*n*	Intervention	Control	*p*	*n*
			*n* = 345	*n* = 152 (75 girls)	*n* = 193 (98 girls)						
			Mean (SD)	Mean (SD)	Mean (SD)			Mean (SD)	Mean (SD)		
Age (years)			6.29 (0.31)	6.29 (0.32)	6.29 (0.30)	0.93	345	6.26 (0.32)^#^	6.3 (0.29)^#^	0.5	187^#^
Family low education (%)^a^			36.8	30.6	41.4	0.05	315	19.1^#^	34.8^#^	0.02	183^#^
Family non-Nordic region (%)^b^			51.3	69.6	37.6	0.00	316	57.4^#^	29.6^#^	0.00	183^#^
Antropometry											
Waist circumference (cm)			57.10 (6.83)	56.43 (6.23)	57.61 (7.24)	0.12	339	56.23 (6.11)	57.68 (7.25)	0.07	307
BMI sds^c^			0.60 (1.35)	0.55 (1.28)	0.65 (1.41)	0.50	342	0.53 (1.27)	0.67 (1.41)	0.37	306
Underweight^d^			4.4	4.6	4.2	0.84	15	3.8	4.0	0.95	12
Normal weight^d^			69.6	70.9	68.6	0.65	238	73.8	67.6	0.23	215
Children with overweight^d^			16.4	15.9	16.8	0.83	56	14.6	17.6	0.48	50
Children with obesity^d^			9.6	8.4	10.5	0.51	33	7.7	10.8	0.36	29
Dietary intake (dl/week)*											
Healthy food			13.59 (8.87)	15.21 (10.57)	12.54 (7.41)	0.10	236	16.15 (9.97)	13.07 (8.18)	0.05	188
Unhealthy food			5.53 (5.36)	5.49 (5.94)	5.56 (4.96)	0.43	236	7.56 (7.01)	7.77 (6.62)	0.85	188
Sweet beverages			6.40 (6.70)	5.75 (5.82)	6.81 (7.20)	0.36	236	6.83 (6.81)	8.52 (8.74)	0.28	188
Physical activity (mins/day)											
MVPA, weekdays			82.66 (24.01)	81.92 (26.57)	83.22 (21.94)	0.65	285	87.41 (24.22)	82.84 (20.19)	0.13	222
MVPA, weekend			62.18 (26.56)	61.28 (27.14)	62.78 (26.25)	0.68	218	63.30 (26.86)	62.92 (28.37)	0.93	157
MVPA, non-school time			21.56 (10.38)	21.43 (12.22)	21.66 (8.78)	0.85	285	22.8 (10.17)	21.49 (7.73)	0.28	222
Sedentary, weekdays			404.49 (55.99)	407.18 (60.83)	402.45 (52.12)	0.48	285	414.22 (54.71)	408.34 (49.63)	0.41	222
Sedentary, weekends			416.20 (72.60)	430.06 (75.18)	406.99 (69.60)	0.02	218	430.40 (75.79)	412.55 (69.74)	0.13	157
Sedentary, non-school time			156.52 (46.76)	156.91 (50.34)	156.23 (43.99)	0.90	285	167.84 (39.65)	162.36 (38.46)	0.30	222

*p* significance level of difference between intervention and control group, *BMI sds* body mass index standard deviation score, *MVPA* moderate to vigorous physical activity. ^a^Lowest reported education level within a family, defined as ≤ 12 years. ^b^One or both parents born outside the Nordic region. ^c^Defined according to Karlberg et al. (2001). ^d^Defined according to Cole and Lobstein (2012). *Based on raw (untransformed) data. LG10 transformed data was used in the analysis. ^#^Sample included in analysis of primary outcome (diet)

**Table 2:** Effects of the intervention on dietary, physical activity, and anthropometric outcomes at T1 in model 1, and unadjusted means at T1

		Model 1							Unadjusted means (SD) at T1 per group			
		*n*	*b*	*p*	95% CI		Between school		Intervention		Control	
Outcomes					Lower	Upper	variance σ_u_^2^ (s.e.)		*n*	Mean (SD)	*n*	Mean (SD)
***Dietary intake (dl/week)***^a^												
Healthy food		188	0.08	0.12	− 0.02	0.18	0.00 (0.00)		71	14.37 (9.20)	117	11.74 (8.81)
Unhealthy food		188	− 0.07	0.17	− 0.17	0.03	0.00 (0.00)		71	4.29 (3.73)	117	5.58 (5.37)
Sweet beverages		188	** − 0.17**	**0.04**	** − 0.33**	** − 0.01**	0.01 (0.01)		71	4.42 (4.67)	117	7.46 (7.49)
***Physical activity (mins/day)***												
MVPA, weekdays^b^		222	**5.68**	**0.02**	**0.8**	**10.56**	0.00 (0.00)		90	91.86 (24.76)	132	83.23 (21.67)
MVPA, weekend^b^		157	2.90	0.64	− 9.89	15.69	68.78 (47.70)		63	72.48 (28.83)	94	67.89 (26.10
MVPA, non-school time^b^		222	1.46	0.29	1.23	4.17	0.00 (0.00)		90	28.20 (12.04)	132	25.64 (12.8)
Sedentary, weekdays^b, c^		222	7.7	0.81	− 1.09	16.47	12.33 (24.39)		90	453.1 (60.49)	132	438.04 (60.93)
Sedentary, weekends^b, c^		157	0.93	0.86	− 9.75	11.61	0.00 (0.00)		63	458.80 (105.86)	94	429.87 (86.86)
Sedentary, non-school time^b,c^		222	7.75	0.12	− 1.9	17.4	0.00 (0.00)		90	199.42 (51.73)	132	182.56 (49.59)
***BMI sds***^d^												
Total sample		306	− 0.03	0.68	− 0.16	0.11	0.00 (0.01)		130	0.46 (1.26)	176	0.61 (1.43)
Children with overweight at T0^e^		50	0.11	0.52	− 0.24	0.46	0.01 (0.03)		19	1.83 (0.48)	31	1.68 (0.66)
Children with obesity at T0^e^		29	− 0.21	0.20	− 0.53	0.11	0.00 (0.00)		10	2.80 (1.05)	19	3.35 (0.82)
***Waist circumference (cm)***												
Total sample		307	0.26	0.65	− 0.94	1.46	0.79 (0.45)		129	57.06 (6.29)	178	58.23 (7.68)
Children with overweight at T0^e^		50	− 0.35	0.83	− 3.73	3.03	4.56 (2.93)		19	61.80 (4.33)	31	62.78 (5.42)
Children with obesity at T0^e^		28	− 0.27	0.89	− 4.52	3.98	1.94 (5.48)		9	70.32 (10.97)	19	74.19 (7.00)

Model 1—adjusted for value of outcome at baseline. Results of Mixed Linear Regression with control group as reference. ^a^Lg10 transformed data were used in the model, ^b^adjusted for monitor wear time, ^c^adjusted for MVPA, ^d^defined according to Karlberg et al., 2001, ^e^defined according to Cole and Lobstein, 2012. *b* regression coefficient (beta), *p* between intervention and control groups, *CI* confidence interval. Healthy food = fruits, vegetables, and legumes. Unhealthy food = crisps, salty snacks, sweets, chocolate, cakes, biscuits, desserts, and other sweet foods. Sweet beverages = soft drinks, flavoured milk, and fruit juice. *MVPA* moderate to vigorous physical activity. Subjects are dependent observations between T0 and T1

**Table 3:** Effects of the intervention on dietary, physical activity, and anthropometric outcomes at T1 in models 2 and 3

Outcomes		**Model 2**						**Model 3**					
		*n*	*b*	*p*	95% CI		Between school	n	*b*	*p*	95% CI		Between school
					Lower	Upper	Variance *σ*_*u*_^2^ (s.e.)				Lower	Upper	Variance *σ*_*u*_^2^ (s.e.)
***Dietary intake (dl/week)***^a^													
Healthy food		183	0.08	0.13	− 0.02	0.17	0.00 (0.00)	183	**0.11**	**0.04**	**0.00**	**0.21**	0.00 (0.00)
Unhealthy food		183	− 0.08	0.12	− 0.19	0.02	0.00 (0.00)	183	− 0.08	0.16	− 0.18	0.03	0.00 (0.00)
Sweet beverages		183	− 0.15	0.07	− 0.32	0.01	0.01 (0.01)	183	− 0.13	0.12	− 0.30	0.04	0.01 (0.01)
***Physical activity (mins/day)***													
MVPA, weekdays^b^		211	**5.05**	**0.046**	**0.1**	**10.01**	0.00 (0.00)	211	**5.77**	**0.047**	**0.07**	**11.47**	8.41 (16.60)
MVPA, weekend^b^		151	2.28	0.71	− 10.49	15.05	59.64 (46.13)	151	7.09	0.20	− 4.14	18.32	26.27 (35.71)
MVPA, non-school time^b^		211	1.43	0.32	− 1.38	4.25	0.00 (0.00)	211	2.28	0.12	− 0.63	5.19	0.00 (0.00)
Sedentary, weekdays^b, c^		211	7.18	0.11	− 1.96	16.31	15.72 (25.6)	211	3.97	0.41	− 6.09	14.62	29.22 (31.05)
Sedentary, weekends^b, c^		151	− 9.56	0.13	− 21.85	2.74	0.00 (0.00)	151	0.15	0.98	− 11.12	11.41	0.00 (0.00)
Low education		47	**23.04**	**0.049**	**0.10**	**45.98**	0.00 (0.00)	NA	NA	NA	NA	NA	NA
High education		104	− 9.51	0.12	− 21.62	2.60	0.00 (0.00)	NA	NA	NA	NA	NA	NA
Sedentary, non-school time^b, c^		211	5.13	0.32	− 4.93	15.19	0.00 (0.00)	211	5.55	0.3	− 4.94	12.97	0.00 (0.00)
***BMI sds***^d^													
Total sample		281	− 0.01	0.90	− 0.13	0.12	0.00 (0.00)	282	0.00	0.95	− 0.12	0.13	0.00 (0.00)
Children with overweight at T0^e^		44	0.12	0.47	− 0.21	0.44	0.00 (0.00)	44	0.01	0.95	− 0.34	0.36	0.00 (0.00)
Children with obesity at T0^e^		26	− 0.25	0.15	− 0.61	0.10	0.00 (0.00)	26	− 0.36	0.12	− 0.81	0.10	0.00 (0.00)
***Waist circumference (cm)***													
Total sample		283	0.30	0.60	− 0.89	1.49	0.72 (0.44)	284	0.16	0.79	− 1.05	1.36	0.72 (0.44)
Children with overweight at T0^e^		44	0.59	0.70	− 2.74	3.92	4.00 (2.77)	44	− 0.12	0.94	− 3.30	3.06	3.49 (2.46)
Children with obesity at T0^e^		25	− 0.52	0.81	− 5.00	3.95	0.55 (4.69)	25	− 1.86	0.47	− 7.07	3.35	2.98 (5.27)

Model 2—adjusted for value of outcome at baseline, child sex and parental education. Model 3—adjusted for value of outcome at baseline child sex and parental region of birth. Results of Mixed Linear Regression with control group as reference. ^a^Lg10 transformed data were used in the model, ^b^adjusted for monitor wear time, ^c^adjusted for MVPA, ^d^defined according to Karlberg et al., 2001, and ^e^defined according to Cole et al. 2012. *b* regression coefficient (beta), *p* between intervention and control groups, *CI* confidence interval. Healthy food = fruits, vegetables, and legumes. Unhealthy food = crisps, salty snacks, sweets, chocolate, cakes, biscuits, desserts, and other sweet foods. Sweet beverages = soft drinks, flavoured milk, and fruit juice. *MVPA* moderate to vigorous physical activity. Subjects are dependent observations between T0 and T1

#### Diet

Dietary data at both T0 and T1 were provided by 188 families, corresponding to a response rate of 53%. These families were more likely to have a higher level of education and be classified as Nordic compared to the whole sample. In the ITT analyses, model 1 showed a significant effect on the intake of sweet beverages (*b* =  − 0.17, *p* = 0.04 CI: − 0.33; − 0.01), whereby the intervention group had a lower intake at T1 compared to the control group (Table 2). In models 2 and 3, the effect on sweet beverages favouring the intervention was no longer significant, but in the same direction and of a similar magnitude (Table 3). In model 3, the intervention group had a higher intake of healthy foods compared to the control group (*b* = 0.11, *p* = 0.04, CI: 0.00; 0.21) (Table 3). In the per protocol analyses, results showed effects in the same direction as the ITT analyses for all three outcomes but were non-significant. In the sensitivity analyses using LVCF, the intervention effect regarding sweet beverages (model 1) remained significant, whereas the effect on healthy foods (model 3) was in the same direction as in the ITT analyses but was non-significant.

#### Physical Activity and Time Spent Sedentary

The requirement of at least 2 days of valid accelerometer data was fulfilled by 263 children (88.0%) at T0 and by 222 children (74.2%) at T1. At T0, 222 children (84.4%) reached the physical activity guidelines of 60 min MVPA. In the ITT analyses, model 1 showed an effect (*b* = 5.68, *p* = 0.02, CI: 0.8; 10.56) on MVPA during weekdays whereby the intervention group had 5 more minutes of MVPA than the control group (Table 2). In model 2, the same significant effect on MVPA was found (*b* = 5.05, *p* = 0.046, CI: 0.1; 10.01, Table 3). Here, an interaction effect with parental education regarding sedentary time during weekends was found (*b* = 23.04, *p* = 0.049, CI: 0.10; 45.98), whereby children with parents with low education in the intervention group had 23 more minutes of sedentary time compared to the control group (Table 3). In model 3, a beneficial effect on MVPA on weekdays (*b* = 5.77, *p* = 0.047, CI: 0.07; 11.47) was detected (Table 3). In the per protocol analyses, results showed intervention effects in the same direction and the effect remained significant, for all outcomes. In the sensitivity analyses using LVCF, intervention effects on MVPA during weekdays in all three models, and sedentary time on weekends for children of parents with low education (model 2) remained significant.

#### Anthropometry

At T0, measurements of height and weight were obtained for 342 (97%) children and waist circumference for 339 (96%). At T1, valid measurements of height and weight were obtained for 315 (89%) children, and waist circumference for 318 (90%). The prevalence of obesity at T0 was 10% (Table 1). In the ITT analyses, no significant intervention effects were detected on either BMI-sds or waist circumference in any overweight category (overweight or obesity) (Tables 2 and 3). In the per protocol analyses, an intervention effect on BMI-sds was found for children in the total sample (*n* = 131) with parents born outside of the Nordic region (*b* =  − 0.16, *p* = 0.02) in model 3.

### Process Evaluation

#### Motivational Interviewing

Of the 155 intervention families, 111 (72%) participated in an MI session. The intervention group’s seven school nurses conducted a median of 14 sessions (range: 2–28). The group mean for the technical component was 1.81 (SD: 0.67), and for the relational component 2.11 (SD: 0.80), which was below the conventional threshold for MI quality (fair = 3.0 (Moyers et al., 2016)). The group mean for the reflection/question ratio 0.92 (SD: 0.47) was close to the threshold (1.0 (Moyers et al., 2016)). The group mean for overall motivational interviewing adherent (MIA) behaviour (1.89 SD: 2.23) was higher than the group mean for motivational interviewing non-adherent (MINA) behaviour (1.66 SD: 2.19), which is desirable in MI sessions. The test on differences in MI competence between intervention and control group based on sessions with a standardised parent character using an actor showed that intervention group nurses had a significantly higher mean score for MIA (p = 0.01) and lower score for MINA (0.001) after MI training compared to control group nurses (who did not receive training). Intervention group nurses also had a higher, but non-significant, mean score on the technical and relational components and the reflection/question ratio.

#### Health Information

Of the parents who participated in the MI sessions (*n* = 111), 41% reported that they had received the brochure, but only 23% reported that they had read the brochure.

#### Classroom Lessons

The mean number of completed lessons (of a potential total of nine) in the 13 classrooms was 8 (SD 2.0), with a median of 9 (range 2–9). The average time spent per lesson was 58 min (SD 9.8), and 89% of the lesson’s core activities had been completed. Regarding the home assignments, 99% had been sent home and of those, 74% had been handed in by the children and subsequently been discussed in class.

#### T2D Risk Test

Parents from 38 intervention families completed the T2D test (25%). In 23 families, both parents completed the test, while only one of the parents did so in 15 of the families*.*

Fidelity to the implementation strategies was generally high. All schools signed a written agreement with the research team, received the written material, and all school nurses in the intervention group received the MI training. A website with all materials for the families and school staff was created, and six out of the eight intervention schools conducted a teacher-led kick-off meeting to engage the parents and prepare collaboration between the school staff and parents.

## Discussion

In this trial, carried out in schools in disadvantaged areas in mid-Sweden, the intervention showed significant beneficial effects on children’s dietary and physical activity behaviours. After the intervention, children in the intervention group had a lower intake of sweet beverages, a higher intake of healthy food, and more minutes in MVPA during weekdays overall compared to the control group. However, an unexpected negative intervention effect was found for sedentary time during weekends for children in families with low education. The evidence for interventions leading to a reduction in socioeconomic inequalities in obesity-related outcomes among children is limited (Hillier-Brown et al., 2014). Research on the effectiveness of interventions among socially disadvantaged children in Europe has shown modest effects on obesity (Wijtzes et al., 2017), diet, and physical activity (Olstad et al., 2017; Pastor and Tur, 2020). Therefore, the effects reported here, from disadvantaged areas, are encouraging and in line with the literature, although it does not necessarily mean that social inequalities have been reduced by this intervention. Future studies with larger, representative samples are needed to determine if social inequalities can be reduced by this approach.

### Dietary Intake

The effects of the HSSP intervention on diet are in line with the previous two trials of the HSS intervention (Nyberg et al., 2015, 2016), where beneficial intervention effects on vegetable intake (Nyberg et al., 2015) and intake of unhealthy foods and unhealthy beverages were found (Nyberg et al., 2016). The estimates in this study correspond to differences of 0.32 dl of sweet beverages and 0.27 dl of healthy foods per week. It is encouraging that the HSSP trial produced similar results to our previous studies because the MI counselling in this trial was carried out by school nurses and not, as in the first two trials, by external qualified MI counsellors who reached higher MI quality. Another Swedish child obesity prevention study using nurse delivered MI counselling also showed small but significant intervention effects in terms of healthier food habits among 4-year-old children and their mothers, while no significant effect on BMI was found (Döring et al., 2016). A systematic review including ten studies which combined dietary and physical activity interventions with and without a caregiver component found a small positive impact of the caregiver component on children’s intake of sweet beverages, in line with our results (Morgan et al., 2020).

### Physical Activity

The HSSP intervention showed a significant effect on MVPA where the intervention group had 5–6 more minutes in MVPA during weekdays. A systematic review found that physical activity interventions had small effects on MVPA in children showing an increase of on average 4 min per day (Metcalf et al., 2012). A meta-analysis of 19 family interventions reached a similar conclusion; the results showed small but significant effects on physical activity in favour of the intervention group (Brown et al., 2016). In contrast, a systematic review found that interventions targeting the school but not the home environment had little to no effect on MVPA with on average about 1 min increase per day in children and adolescents (Neil-Sztramko et al., 2021). In line with the present study, findings from a Finnish family–based lifestyle intervention in children aged 6–8 years showed that parent-reported physical activity increased by 9 min/day in the intervention group and decreased by 5 min/day in the control group (Viitasalo et al., 2016). In addition, the intervention attenuated an increase in sedentary behaviour. The 2-year intervention consisted of 12 counselling sessions and factsheets for the parents, and the children were encouraged to participate in after-school exercise clubs and received financial support for their physical activity. Thus, the Finnish programme was much more intensive than the HSSP where we saw no beneficial intervention effect on sedentary time. On the contrary, the children from parents with low education had 23 more minutes of sedentary time during weekends in the intervention group. One explanation could be that the intervention mainly focused on increasing physical activity and not so much on decreasing sedentary time. More parental involvement might be required for a positive intervention effect on sedentary behaviours, which has also been suggested in a systematic review (Marsh et al., 2014). These authors concluded that the level of parental involvement was important for the success of interventions on sedentary behaviours. The beneficial effects on MVPA in this study may be due to the fact that children are in a period of life when their health behaviours are still very much under the influence of their parents.

### Body Composition

No significant intervention effects were detected on either BMI-sds or waist circumference in the total sample. In the per protocol analysis, a beneficial effect of intervention on BMI-sds was found for children with parents born outside of the Nordic region. Since this is a universal, preventive intervention where 70% of the children are in the normal weight range and 4–5% are underweight, large reductions in BMI-sds on the entire group are neither expected nor desirable. Although non-significant, a reduction in BMI-sds in all three models in children with obesity at baseline is in line with results from the second HSS trial where a significant effect on BMI-sds was found in children with obesity (Nyberg et al., 2016). A pooled analysis of all three HSS trials has confirmed a significant reduction in BMI-sds (− 0.21 BMI-sds) in children with obesity by the HSS programme (Patterson et al., 2024) supporting the observed beneficial effects on various indicators of diet and physical activity (Nyberg et al., 2015, 2016). This is also in agreement with a recent systematic review concluding that strategies for changing children’s diet or physical activity are effective in bringing about modest reductions in BMI *z*-score in children aged 0 to 12 years (Brown et al., 2019). The findings from the HSS trials are encouraging as they suggest that obesity can be reduced by a relatively low-intensive intervention in the school context. However, from previous trials, we know that effects wear off with time, and therefore intervention efforts need to be continued throughout the school years to have sustained effects. Building on these three trials, the HSSP intervention is currently being scaled up and implemented in all public schools in three municipalities in the Stockholm Region and evaluated in a 4-year cluster-randomised comparative hybrid type 3 trial to evaluate the most effective strategies for successful implementation of the HSSP, while monitoring child weight development and parent’s risk of type 2 diabetes (Elinder et al., 2021). By implementing the HSS in several schools within a municipality, staff from different schools can collaborate with each other and with the central education administration to reinforce school health promotion in the municipality. As this also covers older children, it increases the likelihood of sustaining intervention effects. Efforts are also ongoing to combine the universal HSS with a targeted obesity treatment programme developed in Sweden for children with higher needs.

### Fidelity to the Intervention Components

Participation in the MI sessions was 72%, which we consider to be high. Likewise, fidelity to the classroom component can be considered high with a high mean completion rate of both lessons and home assignments. The classroom component targeted child and parent motivation, self-efficacy, and behaviour and is likely to have influenced outcomes in this study. In contrast, adherence to the T2D risk test and the health brochure components can be considered low to medium as only 25% completed the test and less than 50% reported having read the brochure. Thus, the effects of the HSSP are likely explained mainly by the classroom and the MI component, as these also target behaviour more intensely. Efforts will be made to improve fidelity to all components by adjusting the implementation strategies to get parents to read the health brochure and perform the T2D risk test.

Regarding quality of MI delivery, intervention group nurses fell below the conventional threshold for MI quality on two important MI components (technical and relational) and were close to the threshold on one component (reflection/question ratio). Otherwise, they had scored well regarding overall MI-adherent behaviour. Although the intervention group nurses did not exhibit MI competence above the conventional threshold, they clearly adhered more to MI behaviour compared to the control group nurses. After receiving MI training, the intervention group nurses scored higher on all MI quality scores compared to the control group nurses (who had not received training), with significant differences regarding MI-adherent and non-adherent behaviour. Also, it is important to note that the conventional thresholds for MI quality are based on expert opinion, where empirical support is yet to be developed (Moyers et al., 2016). Furthermore, the conventional thresholds focus on MI performed in treatment situations with clients with an established problem in need of change. In the HSSP study, nurses performed MI in a health promotion and prevention context. It has been proposed that such contexts require an adjusted manner of performing MI (Moberg et al., 2021; Norman, 2016). In a health promotion context, lower threshold levels may be sufficient to produce effects on outcomes, as the results of this study would seem to support. As little is known about the required level of MI quality to produce effects on outcomes, especially in prevention settings, this will be investigated in a forthcoming study based on the HSSP data. In the previous HSS trials, external MI counsellors performed the MI sessions, showing higher MI quality, but with similar intervention effects. Also, employing external MI personnel is not a realistic option in intervention scale-up for a number of reasons. In addition, school nurses know the children and families and can provide a more personal approach in reaching out to parents, which is a great benefit in disadvantaged communities.

### Strengths and Limitations

The sample size can be considered a limitation where the total number of participants (*n* = 353) exceeded the sample size required as indicated by the power calculation but where less than 200 families could be included in the analyses of the primary outcome due to missing data. This lack of power increases the likelihood that other, or smaller, differences did not reach the threshold of statistical significance. Regarding the secondary outcomes, the sample size can be considered sufficient to detect relevant effects on physical activity, based on power calculations used in the previous HSS trials (Nyberg et al., 2015, 2016), whereas the study can be considered underpowered to detect group differences in BMI, based on similar studies (Döring et al., 2016). Furthermore, in this study, conducted in disadvantaged areas, we reached families with a somewhat higher than average level of education, which could mean that the results are not completely generalizable to the whole population in these areas. The proportion of parents with post-secondary (university) education in the trial was 73% which is high compared to the average proportion of parents with children in the participating schools with post-secondary education which was 37%. In this trial, the proportion of parents born outside of the Nordic countries was 51%, which is comparable to the proportion of parents in the included schools who were born outside of Sweden (47%). Thus, the trial seems to have reached families with a non-Nordic background to a higher extent than families with low education. The differences in parents’ region of birth between the intervention and control group may be because families of certain nationalities tend to cluster geographically. Also, the eligible number of participants who consented to participate in the study can be considered low despite extensive recruitment strategies including translated information and face-to-face or telephone contact with parents. Additional recruitment strategies, such as more extended face-to-face contact, and networking through community-based organisations should be considered in future health promotion trials in order to capture a more diverse sample of participants which would increase the power of the study and the possibility to generalise the results to a higher extent.

The dietary assessment with a photo-based method is a strength as it can be regarded a semi-objective method, validated in advance (Norman et al., 2020). The high attrition rate in participants performing the dietary method at both T0 and T1 is a weakness; however, the method was more acceptable to parents than a traditional diet questionnaire which was also included but results are not reported as it was not used to measure the primary outcome. Families performing the dietary assessment were more likely to have a higher level of education and be classified as Nordic compared to the entire sample, again limiting the generalisability of the results. A strength of the study is the use of an objective method to measure physical activity and sedentary time. However, accelerometers cannot register for example swimming and cycling, which are popular activities among children. The health brochure and the T2D risk test were not implemented to a satisfactory degree, and more effective implementation strategies might be needed to increase fidelity to these components. The 12-month follow-up data are not presented here but will be reported in a forthcoming paper.

## Conclusion

This study showed that the third iteration of the Healthy School Start intervention had beneficial effects on dietary behaviours and physical activity of children from disadvantaged areas attending their first year of school. The overall effect can be summarised as modest, beneficial changes in the intake of healthy foods and sweet beverages and in MVPA. These results align with the previous two trials of the programme and suggest that this intervention, based on family support in the school setting in disadvantaged areas, is promising and could be scaled up with the potential to decrease social inequalities in health. However, the implementation strategies should be adjusted to achieve higher fidelity to the components carried out by parents. Future research should explore the clinically applicable level of MI required to be effective in health promotion and prevention studies.

## Supplementary Information

Below is the link to the electronic supplementary material.

Supplementary file1 (DOC 219 KB)

Supplementary file2 (DOCX 31 KB)

## Data Availability

The datasets generated and/or analysed during the current study are not publicly available because of ethical reasons, where public availability would compromise participant privacy, but are available from the corresponding author on reasonable request.
